# Adaptive evolution of the osmoregulation-related genes in cetaceans during secondary aquatic adaptation

**DOI:** 10.1186/1471-2148-13-189

**Published:** 2013-09-09

**Authors:** Shixia Xu, Yunxia Yang, Xuming Zhou, Junxiao Xu, Kaiya Zhou, Guang Yang

**Affiliations:** 1 Jiangsu Key Laboratory for Biodiversity and Biotechnology, College of Life Sciences, Nanjing Normal University, 1 Wenyuan Road, Nanjing 210023, China

**Keywords:** Adaptive evolution, Cetaceans, Osmoregulation, Positive selection

## Abstract

**Background:**

Osmoregulation was a primary challenge for cetaceans during the evolutionary transition from a terrestrial to a mainly hyperosmotic environment. Several physiological mechanisms have been suggested to maintain the water and salt balance in cetaceans, but their genetic and evolutionary bases remain poorly explored. The current study investigated the genes involved in osmoregulation in cetaceans and compared them with their counterparts in terrestrial mammals to test whether adaptive evolution occurred during secondary aquatic adaptation.

**Results:**

The present study analyzed the molecular evolution of 11 osmoregulation-related genes in 11 cetacean species, which represented all of the major cetacean clades. The results demonstrated positive selection acting on angiotensin converting enzyme (ACE), angiotensinogen (AGT), SLC14A2, and aquaporin 2 (AQP2). This evidence for the positive selection of AQP2 and SLC14A2 suggests that the adaptive evolution of these genes has helped to enhance the capacity for water and urea transport, thereby leading to the concentration of urine, which is an efficient mechanism for maintaining the water balance. By contrast, a series of positively selected amino acid residues identified in the ACE and AGT (two key members of the renin-angiotensin-aldosterone system, RAAS) proteins of cetaceans suggests that RAAS might have been adapted to maintain the water and salt balance in response to a hyperosmotic environment. Radical amino acid changes in positively selected sites were distributed among most internal and terminal branches of the cetacean phylogeny, which suggests the pervasively adaptive evolution of osmoregulation since the origin of cetaceans and their subsequent diversification.

**Conclusions:**

This is the first comprehensive analysis of the molecular evolution of osmoregulation-related genes in cetaceans in response to selection pressure from a generally hyperosmotic environment. Four genes, i.e., AQP2, SLC14A2, ACE, and AGT were subject to positive selection in cetaceans, which suggests that cetaceans may have adapted to maintain their water and salt balance. This also suggests that cetaceans may have evolved an effective and complex mechanism for osmoregulation.

## Background

Cetaceans are a highly specialized order of mammals that “returned” from the land to the sea approximately 53–56 million years ago (Ma) and they ultimately became a successful and highly diverse group of fully aquatic mammals [1]. During this evolutionary transition from land to generally high salt waters, the maintenance of homeostasis via the regulation of water and electrolyte levels was one of the most critical challenges they encountered.

Marine mammal osmoregulation has been investigated for over a century, but it is still not clear how they developed mechanisms to conserve fresh water and to avoid dehydration. The acquisition of fresh water by cetaceans may occur via seawater consumption, water from food, and water derived from metabolism [2,3]. Compared with terrestrial mammals, marine mammals such as cetaceans consume a water-rich diet of fishes and marine invertebrates [2]. Thus, the water found in their prey and the water derived subsequently from vigorous metabolism are considered to be their primary sources of fresh water [2-4]. In addition, recent studies present evidence that cetaceans acquire fresh water from sea-water because they excrete higher Na^+^ levels than those found in sea-water, especially during fasting periods [5-7]. Moreover, physiological studies show that the water conservation capacity of cetaceans may be better than that of their terrestrial relatives [8,9].

Another primary osmotic challenge for cetaceans is the excretion of the excess salt (Na^+^) ingested with their prey and sea-water. Anatomical evidence suggests that the ‘specialized’ kidneys of cetaceans might be important for salt excretion and water conservation [4,10]. Cetacean kidneys are relatively large compared with those of other mammals and they contain many small, independently functional renules [11,12]. Each renule is similar to those found in other mammals [12,13]. In addition, the relatively large ratio of medulla to renal cortex in cetaceans [11] allows them to produce highly concentrated urine because of enhanced water reabsorption [12]. Thus, the reniculate kidney and increased medullary thickness may be a highly efficient system for salt excretion and water conservation, which represents an evolutionary response to a hyperosmotic environment [14-16].

The hormonal regulation of the salt and water balance is another efficient mechanism in marine mammals, which is also present in terrestrial mammals (reviewed in [3]). Five primary hormones, i.e., angiotensin (ANG, including ANG I, II, or III), renin, aldosterone, atrial natriuretic peptide (ANP), and vasopressin (AVP), are responsible for regulating the salt and water balance in the kidneys of mammals [4]. Three of these, i.e., renin (REN), ANG, and aldosterone, operate via the renin-angiotensin-aldosterone system (RAAS) to modulate the activity of Na^+^-K^+^-ATPase and to increase water reabsorption by mammals [17,18]. RAAS is known to be present in cetaceans and it appears to regulate the salt balance [4,19]. ANP is also responsible for regulating the salt balance by inhibiting Na^+^ transport in the inner medullary collecting ducts [20], although this has not been bioassayed in cetaceans. AVP also contributes significantly to water retention by increasing water reabsorption in the renal collecting ducts [21], whereas large increases in water reabsorption rely heavily on AVP-regulated aquaporins (AQPs), which are responsible for water transport [22,23]. In cetaceans, however, an early study detected no association between the urine flow rate and the plasma AVP concentration in fasting dolphins [24].

Several physiological adaptations for water conservation and salt excretion have been suggested in cetaceans that may have facilitated their adaptation to a high salt environment, but the molecular bases that underlie these mechanisms remain poorly explored. Many genes have been identified that are involved with osmoregulation. For example, Na^+^-K^+^-ATPase, an important salt regulating system, is crucial for maintaining intracellular homeostasis but it also provides the driving force for ion transport in a variety of osmoregulatory epithelia [25]. Na^+^-K^+^-ATPase is a heterodimer with a catalytic α-subunit and a glycosylated β-subunit [26]. The α1 subunit, one of four isoforms (α1-α4), may have a housekeeping role in maintaining the osmotic balance and in regulating the cell-volume [27]. AQPs are important membrane channels that regulate the water balance in the body [28,29]. In particular, some AQPs, such as AQP1–AQP4, are specialized for water re-absorption in the kidney, which has an indispensable role in urine concentration [30]. The increased water re-absorption due to AQPs is heavily reliant on AVP stimulation [21].The urea transporters (UTs) that are highly expressed in the mammalian kidney may also be effective mechanisms for urine concentration [31]. Two genes (SLC14A2 and SLC14A1) encode urea transporters in the mammalian kidney (NCBI http://www.ncbi.nlm.niv.gov). Thus, the renal UTs in mammals act together with the renal AQPs and have integral roles in regulating the body water balance [31]. The present study investigated the coding regions of the Na^+^-K^+^-ATPase α1 subunit, AQP 1–4, SLC14A2, SLC14A1, RAAS (including REN, AGT, and ACE), ANP, and AVP genes in representative cetacean lineages. The genes of cetaceans were compared with those of terrestrial mammals to determine whether they had evolved adaptively during the cetacean transition from the land to the sea, and during their subsequent radiation into waters throughout the world.

## Results

The 10 osmoregulation-related genes examined in this study were sequenced successfully in 11 cetacean species. All genes were intact and there were no premature stop codons or frame-shift mutations, which indicated the presence of functional proteins in cetaceans. Unfortunately, the AVP gene was not amplified successfully in all 11 cetacean species, although various experimental optimizations were attempted. Thus, only the cetacean AVP sequence derived from the baiji (*Lipotes vexillifer*) genome (data unpublished) was used to test selection in the following analysis.

### Molecular evolution of osmoregulatory genes in cetaceans

A pair of site models (M8a vs M8) [32,33] was used to test whether specific codons in the osmoregulatory genes had been subjected to positive selection. Likelihood ratio tests (LRTs) showed that the model incorporating selection (M8) fitted significantly better than the neutral model (M8a) for ACE, REN, and SLC14A2 (Table 1), whereas no significant evidence of positive selection was found for the other eight genes. In model 8, the most stringent model implemented in PAML, a small proportion of codons (0.968%, 1.720%, and 2.207%) were estimated to be under selection, with ω values of 2.077, 16.110, and 1.529 for the ACE, REN, and SLC14A2 genes, respectively. In addition, six, four, and eight sites were identified by the BEB approach as having posterior probabilities ≥ 0.80.

**Table 1:** CODEML analysis of osmoregulatory genes (11 genes) and evidence of positive selection on the ACE, AGT, SLC14A2, REN, AQP2, and AVP genes in mammals and cetaceans

**Genes**	**Models**	**-lnL**	**2ΔLnL**	***P *value**^**a**^	**ω values**
ACE	Site model				
(1033aa)	Dataset I: All mammals (28 sequences)				
	M8a	20856.619			ω = 1
	M8	20853.691	5.856	0.016	ω = 2.077
	Dataset II: cetaceans (11 sequences)				
	M8a	5910.391			ω = 1
	M8	5905.151	10.480	0.001	ω = 4.855
	Branch site model				
	Dataset I: All mammals (28 sequences)				
	Branch n (terminal branch of *Grampus griseus*)				
	Null	21017.590			ω0 = 0.070, ω1 = 1, ω2 = 1
	Alternative	21010.004	15.172	0.002	ω0 = 0.070, ω1 = 1, ω2 = 258.836
AGT	Site model				
(324aa)	Dataset II: cetaceans (10 sequences)				
	M8a	2017.713			ω = 1
	M8	2014.489	6.448	0.011	ω = 4.134
	Branch site model				
	Dataset I: All mammals (30 sequences)				
	Branch n (terminal branch of *G. griseus*)				
	Null	10615.771			ω0 = 0.154, ω1 = 1, ω2 = 1
	Alternative	10601.717	28.107	<0.001	ω0 = 0.154, ω1 = 1, ω2 = 952.146
SLC14A2	Site model				
(861aa)	Dataset I: All mammals (28 sequences)				
	M8a	15199.892			ω = 1
	M8	15197.706	4.372	0.037	ω = 1.529
	Dataset II: cetaceans (10 sequences)				
	M8a	4602.652			ω = 1
	M8	4597.947	9.410	0.002	ω = 2
REN	Site model				
(407aa)	Dataset I: All mammals (25 sequences)				
	M8a	7542.944			ω = 1
	M8	7531.661	22.565	<0.001	ω = 16.110
AQP2	Branch site model				
(202aa)	Dataset I: All mammals (27 sequences)				
	Branch c (ancestral toothed whales)				
	Null	3827.416			ω0 = 0.037, ω1 = 1, ω2 = 1
	Alternative	3822.989	8.854	0.074	ω0 = 0.038, ω1 = 1, ω2 = 999

Note: a, likelihood ratio test p-values were adjusted for multiple testing with a Benjamini & Hochberg’s procedure [63,64] and threshold of 0.05.

Further tests were made to determine whether a similar selection pattern also occurred in cetaceans. ACE and SLC14A2 were found to be under positive selection in the cetacean dataset where the LRTs of the site model were statistically significant (ACE: M8a vs M8: *P* = 0.022; SLC14A2: M8a vs M8: *P* = 0.002); however, unlike the dataset containing all mammals, REN did not show any sign of positive selection (M8a vs M8: *P* = 0.114). By contrast, positive selection was detected in the AGT gene (M8a vs M8: *P* = 0.011). In the M8 model, the average ω values of cetaceans were 4.855, 4.134, and 2.0 for ACE, AGT, and SLC14A2, respectively, and a small proportion of codons (ACE: 1.936% or 20 codons; AGT: 1.852% or six codons; and SLC14A2: 3.136% or 27 codons) were identified as being under positive selection (Table 1). Nine, four, and 19 codons were identified as being positively selected using the BEB approach with posterior probabilities ≥ 0.8 in ACE, AGT, and SLC14A2, respectively.

**Figure 1:**
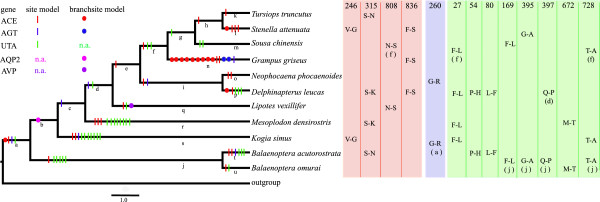
**Radical amino acid changes in selected sites across the cetacean phylogeny.** Parallel amino acid substitutions occurred in three selected genes (ACE, AGT, and SLC14A2) shown on the right of the figure. The selected genes and their corresponding parallel amino acid substitutions in cetaceans are marked with different colors, i.e., ACE (red), AGT (blue), SLC14A2 (green), and AQP2 (purple). Selected sites identified using site models and branch-site models are indicated separately by vertical lines and circles, respectively. For each amino acid position, the parallel amino acid is labeled on the right of the corresponding terminal branches, while a, d, and f in parentheses indicate the internal branches where the parallel changes occurred. Terrestrial mammals were used as outgroups including cetartiodactyls, carnivores, rodents, and primates.

The branch-site model was then used to test for positive selection in individual codons in each lineage of cetaceans (a–u in Figure 1) and the lineages of other groups such as cetartiodactyls, carnivores, rodents, and primates across the phylogeny of all mammals (Additional file 1: Figure S1). The LRT tests showed that there was evidence of positive selection in one cetacean-specific lineage (test 2, *P* < 0.05) after correcting for multiple testing, i.e., the terminal branch of *Grampus griseus* for both ACE (*P* = 0.002) and AGT (*P* < 0.001) (Table 1 and Figure 1). Almost significant positive selection was found at branch b, which led to the last common ancestor of odontocetes for AQP2 (*P* = 0.074) after corrections. Fourteen codons were under positive selection in the two cetacean-specific lineages (Figure 1, Additional file 2: Table S1), whereas no significant signs of positive selection were detected in the lineages of the other groups, i.e., cetartiodactyls, carnivores, rodents, and primates.

Furthermore, 12 positively selective sites were identified using the random effects likelihood (REL) method. Of these putative positively selected sites, 12 (ACE: 158, 246, 373, 315, 792, 808, 836, 911; AGT: 85, 111, 219; AQP2: 105) were detected by both methods whereas only one codon (AGT: 111) was detected by three methods. Thus, four genes (ACE, AGT, SLC14A2, and AQP2) and 45 codons (18 in ACE, five in AGT, 19 in SLC14A2, and three in AQP2) were shown to have been positively selected in cetaceans (Additional file 2: Table S1). The radical amino acid changes in the 45 positively selected codons were scattered throughout most of the cetacean phylogeny (Figure 1). Furthermore, the overwhelming majority of these changes were radical (exact binomial test: *P* < 0.001; Additional file 2: Table S1), which may be additional evidence for positive selection [34-36].

### Spatial distribution of the positively selected sites in the 3D structures

The functional significance of the positively selected sites was investigated further by locating them in the 3D structures of their corresponding genes, which showed that most of the positively selected sites were localized in, or close to, the functional region in the predicted 3D structures of ACE and SLC14A2 (Additional file 3: Figure S2). In the ACE gene, all the 18 positively selected sites were localized in the two zinc-dependent catalytic domains, i.e., 72.222% (13/18) and 27.778% (5/18) in the N and C domains, respectively (Additional file 3: Figure S2). For the SLC14A2 gene, one positively selected site that had undergone radical changes was located in the protein kinase C (PKC) consensus phosphorylation site (Ser-490) in cetaceans.

## Discussion

### Pervasively adaptive evolution of cetacean osmoregulation

Osmoregulation has been studied in marine mammals for over 100 years but there are still no clear conclusions about how their osmoregulation capacity was enhanced in response to a hyperosmotic environment. Most arguments are focused on whether marine mammals can drink sea-water and whether marine mammals can excrete salt-concentrated urine. It is likely that whales ingest sea-water passively while preying on marine animals. In particular, baleen whales filter invertebrates through their baleen plates that are isotonic with seawater so it is inevitable that they will ingest the sea-water in the body fluids of their prey [37]. Furthermore, the ingestion of seawater by cetaceans was confirmed by a recent observation that the Na^+^ concentration of urine is higher than that of sea-water after consuming fish and sea-water [7]. There is still some controversy over whether cetaceans can excrete salt-concentrated urine, but recent studies provide evidence that cetaceans, which are equipped with highly efficient renicules, can produce more concentrated urine than their close terrestrial relatives by concentrating Na^+^ and urea [4,9,38]. By contrast, previous studies have questioned the ability of cetaceans to concentrate urine [39,40].

Most previous studies have focused on the unique renal physiology of marine mammals (reviewed in [3]). Several genes involved with osmoregulation, such as UTs, AQP1, and AQP2, have been cloned in one or two species [40-43], but there is still no comprehensive understanding of the evolution of the osmoregulation-related genes in cetaceans. Thus, the present study conducted comparative analyses of the selective pressure on osmoregulatory genes in cetaceans and terrestrial mammals to test whether adaptive evolution occurred during the origin and diversification of cetaceans. Four lines of evidence support the hypothesis that significantly adaptive molecular evolution of cetacean osmoregulation has occurred. First, the selection analysis showed that four genes involved with osmoregulation, i.e., ACE, AGT, AQP2, and SLC14A2, were subjected to strong positive selection in cetacean-specific lineages, whereas no selection was observed in terrestrial mammalian lineages such as cetartiodactyls, carnivores, rodents, and primates. Second, adaptive evolution was further supported by evidence that radical changes in amino acids have occurred significantly more often than conservative changes in cetacean osmoregulation-related genes. Third, the positively selected sites were localized in, or close to, the functional regions in the predicted 3D structures of the ACE and SLC14A2 genes. Finally, 13 codons (ACE: 246, 315, 808, and 836; AGT: 260; SLC14A2: 27, 54, 80,169, 395, 397, 672, and 728) exhibited parallel amino acid changes in different cetacean lineages (Figure 1). Overall, the significantly positive selection identified in cetaceans suggests an enhancement of their osmoregulation capacity when they shifted their habitat from the land to the sea.

Remarkably, radical changes in amino acids occurred at positively selected sites scattered throughout the entire cetacean phylogeny, from the most common ancestral branch of cetaceans to the terminal branches. This suggests the pervasively adaptive evolution of cetacean osmoregulation. Osmoregulation is a basic requirement of cetaceans that maintains homeostasis in an environment with a high salt concentration so any failure of osmoregulation may lead to an imbalance of water or salt, which may cause further maladaptations in an aquatic environment, possibly causing the death of animals. Thus, it is reasonable to assume that different cetaceans require a fine osmoregulation capacity which has driven the relevant genes to evolve adaptively in response to continuous changes in their osmotic environments since their origin and subsequent diversification in waters throughout the world.

During cetacean evolution, the diversification of river dolphins and their dispersal from sea water to fresh water is a distinct transition. In contrast to their marine counterparts, river dolphins generally inhabit a low-salt (hypoosmotic) habitat that imposes a low osmoregulatory pressure on dolphins because they no longer experience problems conserving fresh water. However, signs of significantly positive selection in ACE, AGT, and SLC14A2 were unexpectedly identified in the baiji lineage, which was the only river dolphin examined in this study. This might be explained by the large difference between the freshwater habitat of river dolphins and that of oceanic dolphins in the sea. This major habitat change presented a new and different osmotic challenge to river dolphins, i.e., maintaining their electrolyte balance, which caused them to modify their osmoregulatory system further as an evolutionary response. To some degree, this may be supported indirectly by the anatomical and physiological differences between freshwater and marine finless porpoises [44].

### Enhanced water, salt, and urea regulation during cetacean osmoregulation

The positive selection on SLC14A2 and AQP2 in the cetaceans considered in the present study supports a previous report that whales can produce higher concentrations of urea in their urine than terrestrial mammals [9]. AQP2 is localized in the collecting tubules/ducts of cetacean renicules, which is also the case in terrestrial mammals, and this protein is essential for the AVP-dependent reabsorption of water [43,45,46]. The positive selection in AQP2 in the cetaceans in the present study suggests an enhanced capacity for water reabsorption in the renal collecting ducts of cetaceans, which could further improve the ability to concentrate salt and urea in the urine. It should be noted that SLC14 A2 knockout mice have a major urinary concentration defect [47], which suggests SLC14A2 plays an important role in maintaining a high concentration of urea. The 19 positively selected sites identified in SLC14A2 in cetaceans also suggest that SLC14A2 must have been adapted to produce high concentration urine in response to a hyperosmotic environment. Furthermore, the positively selected site (490) in the PKC consensus phosphorylation sequence, which caused a radical amino acid change from a polar serine to a nonpolar isoleucine, suggests that whales can concentrate urine by activating PKC and increasing the permeability of urea.

RAAS is responsible for the water and salt balance in mammals [4]. The AGT and ACE genes, which are key members of RAAS, were under positive selection across cetaceans, suggesting cetaceans might have evolved a more effective RAAS to maintain osmoregulation. ANG II is a very powerful regulator of aldosterone release via the adrenal gland in RAAS, which in turn promotes sodium and water reabsorption in the kidneys. ANG II is synthesized via the cleavage of AGT in kidneys, which is mediated by the proteolytic enzyme REN to form ANG I, and this is rapidly converted by ACE to ANG II. Thus, ACE and AGT may be highly important for controlling the formation of ANG II, stimulating aldosterone release, and further regulation of the water and salt balance [18]. This is consistent with previous physiological studies where the plasma concentrations of aldosterone in most cetaceans were higher than those in camels and cattle [9]. By contrast, Na^+^-K^+^-ATPase is a key enzyme involved with direct Na^+^/K^+^ transport, although it is found in many other tissues and is not exclusive to the kidneys. Therefore, no signs of positive selection were detected to indicate significant constraints on this gene, which was probably because of the basic requirement for its function in all tissues. Thus, functional studies of these osmoregulation-related genes are necessary in cetaceans in the future.

### Is widespread positive selection in cetaceans due to the effective population size (*Ne*)?

Population genetic theory predicts that the efficiency of selection should decrease as the effective population size decreses [48]. Thus, selection is relatively ineffective compared with genetic drift and mutation in species with a low *Ne*, and vice versa [49]. In addition, the substitution process is dictated mainly by mutation in species with a low *Ne*. This means that we can expect a higher value of ω with a low population size compared with a high population size. However, three lines of evidence did not support this prediction in the present study. First, the average ω value (ω = 0.22, *d*_N_/*d*_S_ = 0.012/0.056) of the full-length protein in the common cetacean lineage (including the bottlenose dolphin *Tursiops truncatus* and the baiji *L. vexillifer*) was comparable to that in the mouse (*Mus musculus*; ω = 0.14, *d*_N_/*d*_S_ = 0.054/0.39) (unpublished data), considering that smaller mammals tend to have larger effective population sizes [50]. Second, not all cetacean species have a low *Ne*. For example, the effective population size of Pacific gray whales (*Eschrichtius robustus*) has been estimated at between 31,175 and 38,084 breeding adults [51]. Finally, osmoregulation plays a crucial role in cetacean life so even a few mutations could be fatal. Thus, the fixation of mutations in the osmoregulation-related genes of cetaceans must be attributed to selection, which is fairly independent of the effective population size. Thus, all of fixed mutations in cetaceans would increase the overall fitness and adaptive response to hyperosmotic environments.

## Conclusions

The osmoregulation of marine mammals has been investigated for over a century, but its genetic basis remains poorly explored. The present study is one of the first comprehensive analyses of the genetic basis of cetacean osmoregulation. This study detected positive selection particularly in the AQP2, SLC14A2, ACE, and AGT genes of cetaceans, which suggests that cetaceans may have adapted to maintain their water and salt balance in response to a mainly hyperosmotic environment. This also suggests that cetaceans may have evolved an effective and complex mechanism for osmoregulation.

## Methods

### Sequence acquisition and compilation

Eleven cetacean species were sampled, two mysticetes and nine odontocetes (Additional file 4: Table S2). All the cetacean samples used in our study were collected from dead individuals in the wild so that no ethics statement is required. Voucher specimens are preserved at Jiangsu Key Laboratory for Biodiversity and Biotechnology, College of Life Sciences, Nanjing Normal University (NNU), China. The exons of each osmoregulatory gene were sequenced and concatenated, before being analyzed together. Gene sequences from other mammals were downloaded from the OrthoMaM, which is a database that contains orthologous genomic markers for mammals [52]. Only high quality and high integrity sequences were used (the accession numbers for each gene are shown in Additional file 4: Table S2). A total of 47 mammalian species included in 16 orders were analyzed in the present study. The nucleotide and deduced amino acid sequences of each gene were aligned using CLUSTALX 1.83 [53] and MEGA 5.0 [54]. The aligned sequences and phylogenetic trees were deposited in TreeBase (http://purl.org/phylo/treebase/phylows/study/TB2:S14426).

### Selective pressure detection

Selective pressure was tested based on the mammalian phylogeny by comparing the nonsynonymous/synonymous substitution ratios (ω = *d*_N_/*d*_S_) with ω = 1, < 1, and > 1, which indicated neutral evolution, purifying selection, and positive selection, respectively. The ω ratios were estimated using the codon-based maximum likelihood models implemented in the CODEML program in PAML 4.4 [55]. The well-supported phylogeny of Laurasiatheria [56] and primates [57] was used as the input tree in all analyses (Additional file 1: Figure S1). The phylogenetic relationships were also reconstructed using Bayesian inference (BI) for each gene. The resulting trees were similar to the above well-supported phylogeny, with some minor differences in a few branches. Moreover, only the latter analysis is reported because selective detection using the gene tree produced almost identical results to those obtained using the well-supported species phylogeny.

To identify the probabilities of sites under positive selection in each gene, site models were implemented where ω could vary among sites [58,59], which facilitated the analysis of datasets including all mammals or cetaceans only. In particular, a pair of models was tested, i.e., M8a (almost neutral; beta distribution: 0 < ω_0_ < 1 and ω_1_ = 1) vs M8 (positive selection; beta distribution: 0 < ω_0_ < 1 and ω_1_ > 1) [60,61]. Positive selection often operates episodically on a few amino acid sites in a small number of lineages in a phylogenetic tree [62]. Thus, branch-site models were considered in this study that allow ω to vary among sites in the protein and across branches on the tree. Modified branch-site model A (test 2) was performed for each gene in each cetacean lineage across the phylogeny of all mammals. All of the positively selective sites were identified using a Bayes Empirical Bayes (BEB) analysis [55] with posterior probabilities ≥ 0.80. Likelihood ratio test p-values were adjusted for multiple testing with a Benjamini & Hochberg’s procedure [63,64] and threshold of 0.05. In addition, positively selected sites was also inferred using the Random effects likelihood (REL) method implemented in the DATAMONKEY web server [65]. Bayes factor > 50 for REL was implemented. The DATAMONKEY has the advantage that they can improve the estimation of the *d*_N_/*d*_S_ ratio by incorporating variation in the rate of synonymous substitution [65].

The radical amino acid changes that occurred in the positively selected sites were also counted and mapped onto each branch in the cetacean phylogeny. Ancestral sequences for all interior nodes were inferred based on empirical Bayesian methods implemented in the CODEML program in the PAML 4.4 package [55]. Conservative or radical nonsynonymous substitutions were determined according to the methods proposed by Zhang [66].

### Mapping of positively selected sites onto 3D structures

The 3D structures of genes identified as being under positive selection were predicted following the homology modelling using the SWISS-MODEL (http://swissmodel.expasy.org) [67]. The protein sequences of positively selected genes were derived from the *Tursiops truncutus* genome, which were obtained using ENSEMBL (http://www.ensembl.org/index.html). To provide further insights into the functional significance of these positively selected sites, they were mapped onto the 3D structure using PYMOL (http://pymol.sourceforge.net/).

## Abbreviations

RAAS: Renin-angiotensin-aldosterone system; ACE: Angiotensin converting enzyme; AGT: Angiotensinogen; ANG: Angiotensin; ANP: Atrial natriuretic peptide; AVP: Vasopressin; AQPs: Aquaporins; UTs: Urea transporters.

## Competing interests

The authors declare that they have no competing interests.

## Authors’ contributions

SX and GY conceived the project and designed the experiments. YY and XZ performed the experiments, and SX, YY and JX analyzed the data. SX wrote the manuscript, and KZ and GY improve the manuscript. All authors read and approved the final manuscript.

## Supplementary Material

Additional file 1: Figure S1 A well supported phylogeny of mammals used for selective pressure analysis in PAML. Tree topologies of Laurasiatheria and primates were from Zhou et al. [56] and Perelman et al. [57], respectively. Different orders of mammals were marked with different colors. Branches a–u in the tree included in cetaceans are used in the branch-site models tests, and results listed in Table 1 and Figure 1. Click here for file

Additional file 2: Table S1 Radical or conservative changes occurred at positively selected sites detected in cetaceans using the site models and branch-site models. Click here for file

Additional file 3: Figure S2 Spatial distribution of positively selected sites in the three-dimensional (3D) structure of cetacean ACE (A), AGT (B), AQP2 (C), SLC14A2 (D) genes. The 3D structure of ACE and UTA contains two (a1-ACE and a2-ACE) and three domains (d1- SLC14A2, d2- SLC14A2, d3- SLC14A2), respectively. All the positively selected sites were mapped onto 3D structure of each gene. Click here for file

Additional file 4: Table S2 Sequence data used in this study, including taxonomy and accession numbers or Ensembl ID. Click here for file
