# Supplementary material for: Adaptive evolution of the osmoregulation-related genes in cetaceans during secondary aquatic adaptation
Source: BMC Evol Biol. 2013 Sep 9;13:189. doi: 10.1186/1471-2148-13-189 (PMC3848586; doi:10.1186/1471-2148-13-189)
Supplement: Additional file 1: Figure S1 — A well supported phylogeny of mammals used for selective pressure analysis in PAML. Tree topologies of Laurasiatheria and primates were from Zhou et al. [56] and Perelman et al. [57], respectively. Different orders of mammals were marked with different colors. Branches a–u in the tree included in cetaceans are used in the branch-site models tests, and results listed in Table 1 and Figure 1. [file 1471-2148-13-189-S1.doc]

**Additional file 1**

**Figure S1** A well supported phylogeny of mammals used for selective pressure analysis in PAML. Tree topologies of Laurasiatheria and primates were from Zhou et al. [56] and Perelman et al. [57], respectively. Different orders of mammals were marked with different colors. Branches a–u in the tree included in cetaceans are used in the branch-site models tests, and results listed in Table 1and Figure1.
