# Supplementary material for: Adaptive evolution of the osmoregulation-related genes in cetaceans during secondary aquatic adaptation
Source: BMC Evol Biol. 2013 Sep 9;13:189. doi: 10.1186/1471-2148-13-189 (PMC3848586; doi:10.1186/1471-2148-13-189)
Supplement: Additional file 2: Table S1 — Radical or conservative changes occurred at positively selected sites detected in cetaceans using the site models and branch-site models. [file 1471-2148-13-189-S2.doc]

**Additional file 2**

**Table S1** Radical or conservative changes occurred at positively selected sites detected in cetaceans using the site models and branch-site models

| Gene name | AA position | Site model (M8) † | | Branch-site model † | REL(bayes factor>50) | AA change | Radical/conservative change†† | Clades§ |
| --- | --- | --- | --- | --- | --- | --- | --- | --- |
| ACE |  |  | |  |  |  |  |  |
|  | 59 |  | | 0.915 |  | A-S | R | n |
|  | 158 | 0.829 | |  | 72.076 | K-R  N-K | C  R | s,t  a |
|  | 244 |  | | 0.989 |  | T-S | C | n |
|  | 246 | 0.900 | |  | 64.678 | V-G  V-D | R  R | s,l  i |
|  | 247 |  | | 0.943 |  | T-I | R | n |
|  | 249 |  | | 0.940 |  | D-A | R | n |
|  | 253 |  | | 0.993 |  | T-R | R | n |
|  | 256 |  | | 0.939 |  | H-Q | R | n |
|  | 262 |  | | 0.941 |  | Q-H | R | n |
|  | 264 |  | | 0.941 |  | Y-S | R | n |
|  | 265 |  | | 0.941 |  | L-F | R | n |
|  | 313 | 0.905 | |  | 370.027 | T-R  T-K  K-R | R  R  C | j  e  m |
|  | 315 | 0.996 | |  | 396.225 | S-R  S-N  S-K | R  R  R | u  t,k  r,p |
|  | 760 | 0.935 | |  |  | L-F | R | n,o,l |
|  | 792 | 0.811 | |  | 831.129 | N-D  N-S | R  R | t  h |
|  | 808 | 0.962 | |  | 437.429 | N-K  N-S | R  R | s,r  f,q |
|  | 836 | 0.899 | | 0.939 |  | F-S | R | l,n,p |
|  | 911 | 0.837 | |  | 81.950 | Y-R | R | r |
| AGT |  |  |  | |  |  |  |  |
|  | 85 | 0.949 |  | | 1710.420 | K-G  K-E  K-Q | R  R  R | s  t  d |
|  | 111 | 0.913 | 0.981 | | 920.409 | M-V  M-C | C  R | q  n |
|  | 112 |  | 0.984 | |  | L-W | R | n |
|  | 219 | 0.823 |  | | 441.907 | L-M  M-V  M-I  M-L | C  C  C  C | c  r  q  a |
|  | 260 | 0.810 |  | |  | R-G  G-R | R  R | i  a,c |
| SLC14A2 |  |  |  | |  |  |  |  |
|  | 27 | 0.997 |  | |  | F-L | R | f,p,r,s |
|  | 28 | 0.800 |  | |  | T-N | R | r,s |
|  | 54 | 0.805 |  | |  | P-H | R | p,t |
|  | 80 | 0.801 |  | |  | L-F | R | p,t |
|  | 132 | 0.803 |  | |  | M-V | C | r,s |
|  | 169 | 0.808 |  | |  | F-L | R | j,m |
|  | 229 | 0.916 |  | |  | I-V  I-T | C  R | j  s |
|  | 354 | 0.937 |  | |  | T-M  T-A | R  R | j  s,r |
|  | 395 | 0.894 |  | |  | G-A | R | j,g |
|  | 397 | 0.895 |  | |  | Q-P | R | d,j |
|  | 412 | 0.839 |  | |  | V-M | C | s,r |
|  | 468 | 0.828 |  | |  | S-T  S-R | C  R | i  m |
|  | 490 | 0.857 |  | |  | S-I | R | s,r |
|  | 529 | 0.923 |  | |  | T-V | R | t |
|  | 558 | 0.885 |  | |  | S-L | R | s,r |
|  | 618 | 0.971 |  | |  | V-M  M-V | C  C | g,p,r  a |
|  | 664 | 0.894 |  | |  | T-K | R | a,q,r |
|  | 672 | 0.820 |  | |  | M-T  V-M | R  C | u,r  a |
|  | 728 | 0.926 |  | |  | T-A | R | f,j,s |
| AQP2 |  |  |  | |  |  |  |  |
|  | 22 |  |  | | 2198.4 | L-I  L-V | C  C | o,p  m,k,q |
|  | 81 |  | 0.989 | |  | V-I | C | b,i |
|  | 105 |  | 0.993 | | 887.867 | H-S | R | b |

†Posterior probabilities in the BEB analysis where *P* ≥ 0.8 indicated candidates for selection.

††Radical and conservative nonsynonymous substitutions (abbreviated as R and C, respectively), which were estimated according to the methods proposed by Zhang [66].

§Clades where amino acid substitution occurred, with detailed information marked in Figure 1.
