# Supplementary material for: Adaptive evolution of the osmoregulation-related genes in cetaceans during secondary aquatic adaptation
Source: BMC Evol Biol. 2013 Sep 9;13:189. doi: 10.1186/1471-2148-13-189 (PMC3848586; doi:10.1186/1471-2148-13-189)
Supplement: Additional file 3: Figure S2 — Spatial distribution of positively selected sites in the three-dimensional (3D) structure of cetacean ACE (A), AGT (B), AQP2 (C), SLC14A2 (D) genes. The 3D structure of ACE and UTA contains two (a1-ACE and a2-ACE) and three domains (d1- SLC14A2, d2- SLC14A2, d3- SLC14A2), respectively. All the positively selected sites were mapped onto 3D structure of each gene. [file 1471-2148-13-189-S3.doc]

**Additional file 3**

**Figure S2** Spatial distribution of positively selected sites in the three-dimensional (3D) structure of cetacean ACE (A), AGT (B), AQP2 (C), SLC14A2 (D) genes. The 3D structure of ACE and UTA contains two (a1-ACE and a2-ACE) and three domains (d1- SLC14A2, d2- SLC14A2, d3- SLC14A2), respectively. All the positively selected sites were mapped onto 3D structure of each gene.
