# Supplementary material for: Adaptive evolution of the osmoregulation-related genes in cetaceans during secondary aquatic adaptation
Source: BMC Evol Biol. 2013 Sep 9;13:189. doi: 10.1186/1471-2148-13-189 (PMC3848586; doi:10.1186/1471-2148-13-189)
Supplement: Additional file 4: Table S2 — Sequence data used in this study, including taxonomy and accession numbers or Ensembl ID. [file 1471-2148-13-189-S4.doc]

**Additional file 4 Table S2** Sequence data used in this study, including taxonomy and accession numbers or Ensembl ID

| Classification | Species name | Accession numbers | | | | | | | | | | | | |  |  |
| --- | --- | --- | --- | --- | --- | --- | --- | --- | --- | --- | --- | --- | --- | --- | --- | --- |
|  |  | ACE | AQP1 | AQP2 | AQP3 | | AQP4 | | Na+-K+-  ATPase α1 | UTA | UTB | AGT | | REN | ANP | AVP |
| **Superorder Laurasiatheria** | |  |  |  |  | |  | |  |  |  | |  |  |  |  |
| **Cetartiodactyla** | *Grampus griseus* | KC153313 | KC153337 | - | - | | KC153361 | | - | KC153394 | KC153397 | KC153320 | | KC153385 | KC153327 | - |
|  | *Stenela attenuatta* | KC153312 | - | - | - | | - | | - | - | - | - | | - | - | - |
|  | *Sousa chinensis* | KC153311 | KC153336 | KC153348 | KC153352 | | KC153359 | | KC153372 | KC153395 | KC153398 | KC153318 | | KC153386 | KC153326 | - |
|  | *Tursiops truncatus* | KC153314 | KC153335 | KC153350 | KC153351 | | KC153358 | | KC153369 | KC153396 | KC153399 | KC153317 | | KC153377 | KC153325 | - |
|  | *Neophocaena phocaenoides* | KC153309 | - | KC153346 | KC153357 | | KC153364 | | KC153371 | KC153393 | KC153404 | KC153316 | | KC153383 | KC153329 | - |
|  | *Delphinapterus leucas* | KC153310 | KC153342 | KC153349 | - | | KC153362 | | - | KC153392 | KC153405 | KC153319 | | KC153384 | KC153328 | - |
|  | *Lipotes vexillifer* | KC153308 | - | KC153345 | KC153356 | | KC153365 | | KC153374 | KC153391 | KC153400 | KC153315 | | KC153378 | KC153330 |  |
|  | *Mesoplodon densirostris* | KC153307 | KC153340 | KC153344 | KC153353 | | KC153366 | | KC153373 | KC153390 | KC153402 | KC153323 | | KC153380 | KC153331 | - |
|  | *Kogia sima* | KC153306 | KC153341 | - | KC153355 | | KC153367 | | KC153376 | KC153389 | KC153403 | KC153324 | | KC153379 | KC153332 | - |
|  | *Balaenoptera acutorostrata* | KC153304 | KC153339 | KC153347 | KC153354 | | KC153368 | | KC153375 | KC153388 | KC153401 | KC153321 | | KC153381 | KC153333 | - |
|  | *B. omurai* | KC153305 | KC153338 | KC153343 | - | | - | | - | KC153387 | KC153406 | KC153322 | | KC153382 | KC153334 | - |
|  | *Bos taurus* | [ENSBTAG00000024950](http://www.ensembl.org/Bos_taurus/geneview?gene=ENSBTAG00000024950) | [ENSBTAG00000000745](http://www.ensembl.org/Bos_taurus/geneview?gene=ENSBTAG00000000745) | ENSBTAT00000011024 | [ENSBTAG00000008493](http://www.ensembl.org/Bos_taurus/geneview?gene=ENSBTAG00000008493) | | [ENSBTAG00000019037](http://www.ensembl.org/Bos_taurus/geneview?gene=ENSBTAG00000019037) | | [ENSBTAG00000001246](http://www.ensembl.org/Bos_taurus/geneview?gene=ENSBTAG00000001246) | - | [ENSBTAG00000019870](http://www.ensembl.org/Bos_taurus/geneview?gene=ENSBTAG00000019870) | ENSBTAG00000012393  - | | [ENSBTAG00000021333](http://www.ensembl.org/Bos_taurus/geneview?gene=ENSBTAG00000021333) | [ENSBTAG00000006709](http://www.ensembl.org/Bos_taurus/geneview?gene=ENSBTAG00000006709) | [ENSBTAG00000008027](http://www.ensembl.org/Bos_taurus/geneview?gene=ENSBTAG00000008027) |
|  | *Sus scrofa* | [ENSSSCG00000017296](http://www.ensembl.org/Sus_scrofa/geneview?gene=ENSSSCG00000017296) | - | ENSSSCT00000000225 | [ENSSSCG00000010991](http://www.ensembl.org/Sus_scrofa/geneview?gene=ENSSSCG00000010991) | | [ENSSSCG00000003720](http://www.ensembl.org/Sus_scrofa/geneview?gene=ENSSSCG00000003720) | | [ENSSSCG00000006740](http://www.ensembl.org/Sus_scrofa/geneview?gene=ENSSSCG00000006740) | [ENSSSCG00000004491](http://www.ensembl.org/Sus_scrofa/geneview?gene=ENSSSCG00000004491) | [ENSSSCG00000004492](http://www.ensembl.org/Sus_scrofa/geneview?gene=ENSSSCG00000004492) | - | | - | [ENSSSCG00000003430](http://www.ensembl.org/Sus_scrofa/geneview?gene=ENSSSCG00000003430) | [ENSSSCG00000007163](http://www.ensembl.org/Sus_scrofa/geneview?gene=ENSSSCG00000007163) |
|  | *Vicugna pacos* | - | - | ENSVPAG00000007032 | - | | [ENSVPAG00000002693](http://www.ensembl.org/Vicugna_pacos/geneview?gene=ENSVPAG00000002693) | | - | - | [ENSVPAG00000009831](http://www.ensembl.org/Vicugna_pacos/geneview?gene=ENSVPAG00000009831) | - | | - | - | - |
| **Perissodactyla** | *Equus caballus* | - | [ENSECAG00000008979](http://www.ensembl.org/Equus_caballus/geneview?gene=ENSECAG00000008979) | ENSECAG00000023681 | [ENSECAG00000021134](http://www.ensembl.org/Equus_caballus/geneview?gene=ENSECAG00000021134) | | [ENSECAG00000004242](http://www.ensembl.org/Equus_caballus/geneview?gene=ENSECAG00000004242) | | [ENSECAG00000024623](http://www.ensembl.org/Equus_caballus/geneview?gene=ENSECAG00000024623) | [ENSECAG00000021006](http://www.ensembl.org/Equus_caballus/geneview?gene=ENSECAG00000021006) | [ENSECAG00000014487](http://www.ensembl.org/Equus_caballus/geneview?gene=ENSECAG00000014487) |  | | - | [ENSECAG00000014892](http://www.ensembl.org/Equus_caballus/geneview?gene=ENSECAG00000014892) | - |
| **Carnivora** | *Canis familiaris* | [ENSCAFG00000012998](http://www.ensembl.org/Canis_familiaris/geneview?gene=ENSCAFG00000012998) | [ENSCAFG00000003102](http://www.ensembl.org/Canis_familiaris/geneview?gene=ENSCAFG00000003102) | ENSCAFT00000013300 | [ENSCAFG00000023922](http://www.ensembl.org/Canis_familiaris/geneview?gene=ENSCAFG00000023922) | | [ENSCAFG00000018125](http://www.ensembl.org/Canis_familiaris/geneview?gene=ENSCAFG00000018125) | | [ENSCAFG00000009795](http://www.ensembl.org/Canis_familiaris/geneview?gene=ENSCAFG00000009795) | [ENSCAFG00000017672](http://www.ensembl.org/Canis_familiaris/geneview?gene=ENSCAFG00000017672) | [ENSCAFG00000017664](http://www.ensembl.org/Canis_familiaris/geneview?gene=ENSCAFG00000017664) | ENSCAFG00000012068 | | [ENSCAFG00000009623](http://www.ensembl.org/Canis_familiaris/geneview?gene=ENSCAFG00000009623) | - | [ENSCAFG00000006432](http://www.ensembl.org/Canis_familiaris/geneview?gene=ENSCAFG00000006432) |
|  | *Ailuropoda melanoleuca* | [ENSAMEG00000003736](http://www.ensembl.org/Ailuropoda_melanoleuca/geneview?gene=ENSAMEG00000003736) | - | - | - | | - | | - | [ENSAMEG00000005289](http://www.ensembl.org/Ailuropoda_melanoleuca/geneview?gene=ENSAMEG00000005289) | [ENSAMEG00000012521](http://www.ensembl.org/Ailuropoda_melanoleuca/geneview?gene=ENSAMEG00000012521) | ENSAMEG00000009713 | | [ENSAMEG00000000927](http://www.ensembl.org/Ailuropoda_melanoleuca/geneview?gene=ENSAMEG00000000927) | - | - |
|  | *Felis catus* |  | [ENSFCAG00000002542](http://www.ensembl.org/Felis_catus/geneview?gene=ENSFCAG00000002542) | ENSFCAG00000009457 | - | | - | | [ENSFCAG00000013378](http://www.ensembl.org/Felis_catus/geneview?gene=ENSFCAG00000013378) | [ENSFCAG00000010670](http://www.ensembl.org/Felis_catus/geneview?gene=ENSFCAG00000010670) | - | - | | - | [ENSFCAG00000000070](http://www.ensembl.org/Felis_catus/geneview?gene=ENSFCAG00000000070) | - |
| **Chiroptera** | *Pteropus vampyrus* | - | [ENSPVAG00000010577](http://www.ensembl.org/geneview?gene=ENSPVAG00000010577) | ENSPVAG00000006693 | [ENSPVAG00000011486](http://www.ensembl.org/Pteropus_vampyrus/geneview?gene=ENSPVAG00000011486) | | [ENSPVAG00000006692](http://www.ensembl.org/Pteropus_vampyrus/geneview?gene=ENSPVAG00000006692) | | [ENSPVAG00000006284](http://www.ensembl.org/Pteropus_vampyrus/geneview?gene=ENSPVAG00000006284) | - | [ENSPVAG00000014690](http://www.ensembl.org/Pteropus_vampyrus/geneview?gene=ENSPVAG00000014690) | - | | - | [ENSPVAG00000001958](http://www.ensembl.org/Pteropus_vampyrus/geneview?gene=ENSPVAG00000001958) | [ENSPVAG00000014205](http://www.ensembl.org/Pteropus_vampyrus/geneview?gene=ENSPVAG00000014205) |
|  | *Myotis lucifugus* |  | - | - | [ENSMLUG00000012213](http://www.ensembl.org/Myotis_lucifugus/geneview?gene=ENSMLUG00000012213) | | - | | - | [ENSMLUG00000000990](http://www.ensembl.org/Myotis_lucifugus/geneview?gene=ENSMLUG00000000990) | [ENSMLUG00000004885](http://www.ensembl.org/Myotis_lucifugus/geneview?gene=ENSMLUG00000004885) | ENSPVAG00000009216 | | - | - | - |
| **Eulipotyphla** | *Erinaceus europaeus* | - | - | - | [ENSEEUG00000000412](http://www.ensembl.org/Erinaceus_europaeus/geneview?gene=ENSEEUG00000000412) | | - | | [ENSEEUG00000003276](http://www.ensembl.org/Erinaceus_europaeus/geneview?gene=ENSEEUG00000003276) | - | - | - | | [ENSEEUG00000001053](http://www.ensembl.org/Erinaceus_europaeus/geneview?gene=ENSEEUG00000001053) | [ENSEEUG00000007936](http://www.ensembl.org/Erinaceus_europaeus/geneview?gene=ENSEEUG00000007936) | - |
|  | *Sorex araneus* | - | - | - | - | | - | | [ENSSARG00000004972](http://www.ensembl.org/Sorex_araneus/geneview?gene=ENSSARG00000004972) | - | - | - | | - | - | - |
| **Primates** | *Homo sapiens* | [ENSG00000159640](http://www.ensembl.org/Homo_sapiens/geneview?gene=ENSG00000159640) | [ENSG00000240583](http://www.ensembl.org/Homo_sapiens/geneview?gene=ENSG00000240583) | ENSG00000167580 | - | | [ENSG00000171885](http://www.ensembl.org/Homo_sapiens/geneview?gene=ENSG00000171885) | | [ENSG00000163399](http://www.ensembl.org/Homo_sapiens/geneview?gene=ENSG00000163399) | [ENSG00000132874](http://www.ensembl.org/Homo_sapiens/geneview?gene=ENSG00000132874) | [ENSG00000141469](http://www.ensembl.org/Homo_sapiens/geneview?gene=ENSG00000141469) | [ENSG00000135744](http://www.ensembl.org/Homo_sapiens/geneview?gene=ENSG00000135744) | | [ENSG00000143839](http://www.ensembl.org/Homo_sapiens/geneview?gene=ENSG00000143839) | [ENSG00000175206](http://www.ensembl.org/Homo_sapiens/geneview?gene=ENSG00000175206) | [ENSG00000101200](http://www.ensembl.org/Homo_sapiens/geneview?gene=ENSG00000101200) |
|  | *Pan troglodytes* | - | [ENSPTRG00000019047](http://www.ensembl.org/Pan_troglodytes/geneview?gene=ENSPTRG00000019047) | - | [ENSPTRG00000020860](http://www.ensembl.org/Pan_troglodytes/geneview?gene=ENSPTRG00000020860) | | [ENSPTRG00000009936](http://www.ensembl.org/Pan_troglodytes/geneview?gene=ENSPTRG00000009936) | | [ENSPTRG00000001152](http://www.ensembl.org/Pan_troglodytes/geneview?gene=ENSPTRG00000001152) | [ENSPTRG00000009987](http://www.ensembl.org/Pan_troglodytes/geneview?gene=ENSPTRG00000009987) | - | [ENSPTRG00000002096](http://www.ensembl.org/Pan_troglodytes/geneview?gene=ENSPTRG00000002096) | | [ENSPTRG00000001883](http://www.ensembl.org/Pan_troglodytes/geneview?gene=ENSPTRG00000001883) | [ENSPPYG00000001878](http://www.ensembl.org/Pongo_abelii/geneview?gene=ENSPPYG00000001878) | [ENSPTRG00000013190](http://www.ensembl.org/Pan_troglodytes/geneview?gene=ENSPTRG00000013190) |
|  | *Gorilla gorilla* | [ENSGGOG00000000642](http://www.ensembl.org/Gorilla_gorilla/geneview?gene=ENSGGOG00000000642) | [ENSGGOG00000009877](http://www.ensembl.org/Gorilla_gorilla/geneview?gene=ENSGGOG00000009877) | ENSGGOG00000005897 | [ENSGGOG00000003993](http://www.ensembl.org/Gorilla_gorilla/geneview?gene=ENSGGOG00000003993) | | - | | - | [ENSGGOG00000001047](http://www.ensembl.org/Gorilla_gorilla/geneview?gene=ENSGGOG00000001047) | [ENSGGOG00000008152](http://www.ensembl.org/Gorilla_gorilla/geneview?gene=ENSGGOG00000008152) | [ENSGGOG00000000197](http://www.ensembl.org/Gorilla_gorilla/geneview?gene=ENSGGOG00000000197) | | [ENSGGOG00000000840](http://www.ensembl.org/Gorilla_gorilla/geneview?gene=ENSGGOG00000000840) | - | - |
|  | *Pongo pygmaeus* | [ENSPPYG00000008523](http://www.ensembl.org/Pongo_abelii/geneview?gene=ENSPPYG00000008523) | - | ENSPPYG00000004500 | - | | [ENSPPYG00000009079](http://www.ensembl.org/Pongo_abelii/geneview?gene=ENSPPYG00000009079) | | [ENSPPYG00000000985](http://www.ensembl.org/Pongo_abelii/geneview?gene=ENSPPYG00000000985) | [ENSPPYG00000009124](http://www.ensembl.org/Pongo_abelii/geneview?gene=ENSPPYG00000009124) | [ENSPPYG00000009125](http://www.ensembl.org/Pongo_abelii/geneview?gene=ENSPPYG00000009125) | [ENSPPYG00000000120](http://www.ensembl.org/Pongo_abelii/geneview?gene=ENSPPYG00000000120) | | [ENSPPYG00000000322](http://www.ensembl.org/Pongo_abelii/geneview?gene=ENSPPYG00000000322) | - | [ENSPPYG00000010827](http://www.ensembl.org/Pongo_abelii/geneview?gene=ENSPPYG00000010827) |
|  | *Nomascus leucogenys* | - | - | - | - | | - | | - | ENSNLEG00000010799 | ENSNLEG00000010845 | ENSNLEG00000001707 | | ENSNLEG00000014231 | - | - |
|  | *Macaca mulatta* | - | [ENSMMUG00000011763](http://www.ensembl.org/Macaca_mulatta/geneview?gene=ENSMMUG00000011763) | ENSMMUG00000022916 | [ENSMMUG00000003314](http://www.ensembl.org/Macaca_mulatta/geneview?gene=ENSMMUG00000003314) | | [ENSMMUG00000014934](http://www.ensembl.org/Macaca_mulatta/geneview?gene=ENSMMUG00000014934) | | [ENSMMUG00000008151](http://www.ensembl.org/Macaca_mulatta/geneview?gene=ENSMMUG00000008151) | [ENSMMUG00000004010](http://www.ensembl.org/Macaca_mulatta/geneview?gene=ENSMMUG00000004010) | - | [ENSMMUG00000005881](http://www.ensembl.org/Macaca_mulatta/geneview?gene=ENSMMUG00000005881) | | [ENSMMUG00000008464](http://www.ensembl.org/Macaca_mulatta/geneview?gene=ENSMMUG00000008464) | [ENSMMUG00000007834](http://www.ensembl.org/Macaca_mulatta/geneview?gene=ENSMMUG00000007834) | - |
|  | *Callithrix jacchus* | [ENSCJAG00000019755](http://www.ensembl.org/Callithrix_jacchus/geneview?gene=ENSCJAG00000019755) | - | ENSCJAG00000021049 | [ENSCJAG00000008188](http://www.ensembl.org/Callithrix_jacchus/geneview?gene=ENSCJAG00000008188) | | [ENSCJAG00000020734](http://www.ensembl.org/Callithrix_jacchus/geneview?gene=ENSCJAG00000020734) | | [ENSCJAG00000006058](http://www.ensembl.org/Callithrix_jacchus/geneview?gene=ENSCJAG00000006058) | [ENSCJAG00000004051](http://www.ensembl.org/Callithrix_jacchus/geneview?gene=ENSCJAG00000004051) | - | [ENSCJAG00000011972](http://www.ensembl.org/Callithrix_jacchus/geneview?gene=ENSCJAG00000011972) | | [ENSCJAG00000016857](http://www.ensembl.org/Callithrix_jacchus/geneview?gene=ENSCJAG00000016857) | [ENSCJAG00000013443](http://www.ensembl.org/Callithrix_jacchus/geneview?gene=ENSCJAG00000013443) | [ENSCJAG00000021094](http://www.ensembl.org/Callithrix_jacchus/geneview?gene=ENSCJAG00000021094) |
|  | *Otolemur garnettii* | [ENSOGAG00000006842](http://www.ensembl.org/Otolemur_garnettii/geneview?gene=ENSOGAG00000006842) | [ENSOGAG00000001908](http://www.ensembl.org/Otolemur_garnettii/geneview?gene=ENSOGAG00000001908) | - | - | | - | | [ENSOGAG00000000187](http://www.ensembl.org/Otolemur_garnettii/geneview?gene=ENSOGAG00000000187) | [ENSOGAG00000016395](http://www.ensembl.org/Otolemur_garnettii/geneview?gene=ENSOGAG00000016395) | [ENSOGAG00000016407](http://www.ensembl.org/Otolemur_garnettii/geneview?gene=ENSOGAG00000016407) | [ENSOGAG00000011286](http://www.ensembl.org/Otolemur_garnettii/geneview?gene=ENSOGAG00000011286) | | [ENSOGAG00000011896](http://www.ensembl.org/Otolemur_garnettii/geneview?gene=ENSOGAG00000011896) | [ENSOGAG00000009737](http://www.ensembl.org/Otolemur_garnettii/geneview?gene=ENSOGAG00000009737) | - |
|  | *Microcebus murinus* | [ENSMICG00000003785](http://www.ensembl.org/Microcebus_murinus/geneview?gene=ENSMICG00000003785) | [ENSMICG00000012214](http://www.ensembl.org/Microcebus_murinus/geneview?gene=ENSMICG00000012214) | - | [ENSMICG00000012148](http://www.ensembl.org/Microcebus_murinus/geneview?gene=ENSMICG00000012148) | | - | | - | - | - | - | | - | - | - |
|  | *Tupaia belangeri* | - | [ENSTBEG00000003908](http://www.ensembl.org/Tupaia_belangeri/geneview?gene=ENSTBEG00000003908) | - | [ENSTBEG00000003128](http://www.ensembl.org/Tupaia_belangeri/geneview?gene=ENSTBEG00000003128) | | [ENSTBEG00000007931](http://www.ensembl.org/Tupaia_belangeri/geneview?gene=ENSTBEG00000007931) | | - | - | [ENSTBEG00000000797](http://www.ensembl.org/Tupaia_belangeri/geneview?gene=ENSTBEG00000000797) | - | | - | - | - |
| **Rodentia** | *Cavia porcellus* | - | [ENSCPOG00000011393](http://www.ensembl.org/Cavia_porcellus/geneview?gene=ENSCPOG00000011393) | - | [ENSCPOG00000012277](http://www.ensembl.org/Cavia_porcellus/geneview?gene=ENSCPOG00000012277) | | - | | [ENSCPOG00000000927](http://www.ensembl.org/Cavia_porcellus/geneview?gene=ENSCPOG00000000927) | [ENSCPOG00000007976](http://www.ensembl.org/Cavia_porcellus/geneview?gene=ENSCPOG00000007976) | [ENSCPOG00000007979](http://www.ensembl.org/Cavia_porcellus/geneview?gene=ENSCPOG00000007979) | [ENSCPOG00000023212](http://www.ensembl.org/Cavia_porcellus/geneview?gene=ENSCPOG00000023212) | | [ENSCPOG00000022768](http://www.ensembl.org/Cavia_porcellus/geneview?gene=ENSCPOG00000022768) | - | [ENSCPOG00000023592](http://www.ensembl.org/Cavia_porcellus/geneview?gene=ENSCPOG00000023592) |
|  | *Spermophilus tridecemlineatus* | - | - | ENSSTOG00000011259 | - | | - | | - | - | - | - | | - | - | - |
|  | *Dipodomys ordii* | - | - | - | [ENSDORG00000006017](http://www.ensembl.org/Dipodomys_ordii/geneview?gene=ENSDORG00000006017) | | - | | - | -- | - | - | | - | [ENSDORG00000014043](http://www.ensembl.org/Dipodomys_ordii/geneview?gene=ENSDORG00000014043) | - |
|  | *Mus musculus* | - | [ENSMUSG00000004655](http://www.ensembl.org/Mus_musculus/geneview?gene=ENSMUSG00000004655) | ENSMUSG00000023013 | [ENSMUSG00000028435](http://www.ensembl.org/Mus_musculus/geneview?gene=ENSMUSG00000028435) | | [ENSMUSG00000024411](http://www.ensembl.org/Mus_musculus/geneview?gene=ENSMUSG00000024411) | | [ENSMUSG00000033161](http://www.ensembl.org/Mus_musculus/geneview?gene=ENSMUSG00000033161) | [ENSMUSG00000024552](http://www.ensembl.org/Mus_musculus/geneview?gene=ENSMUSG00000024552) | [ENSMUSG00000059336](http://www.ensembl.org/Mus_musculus/geneview?gene=ENSMUSG00000059336) | [ENSMUSG00000031980](http://www.ensembl.org/Mus_musculus/geneview?gene=ENSMUSG00000031980) | | [ENSMUSG00000070645](http://www.ensembl.org/Mus_musculus/geneview?gene=ENSMUSG00000070645) | [ENSMUSG00000041616](http://www.ensembl.org/Mus_musculus/geneview?gene=ENSMUSG00000041616) | [ENSMUSG00000037727](http://www.ensembl.org/Mus_musculus/geneview?gene=ENSMUSG00000037727) |
|  | *Rattus norvegicus* | [ENSRNOG00000007467](http://www.ensembl.org/Rattus_norvegicus/geneview?gene=ENSRNOG00000007467) | [ENSRNOG00000011648](http://www.ensembl.org/Rattus_norvegicus/geneview?gene=ENSRNOG00000011648) | ENSRNOG00000000297 | [ENSRNOG00000009797](http://www.ensembl.org/Rattus_norvegicus/geneview?gene=ENSRNOG00000009797) | | [ENSRNOG00000016043](http://www.ensembl.org/Rattus_norvegicus/geneview?gene=ENSRNOG00000016043) | |  | [ENSRNOG00000016393](http://www.ensembl.org/Rattus_norvegicus/geneview?gene=ENSRNOG00000016393) | [ENSRNOG00000016753](http://www.ensembl.org/Rattus_norvegicus/geneview?gene=ENSRNOG00000016753) | [ENSRNOG00000018445](http://www.ensembl.org/Rattus_norvegicus/geneview?gene=ENSRNOG00000018445) | | [ENSRNOG00000002937](http://www.ensembl.org/Rattus_norvegicus/geneview?gene=ENSRNOG00000002937) | [ENSRNOG00000008176](http://www.ensembl.org/Rattus_norvegicus/geneview?gene=ENSRNOG00000008176) | [ENSRNOG00000021229](http://www.ensembl.org/Rattus_norvegicus/geneview?gene=ENSRNOG00000021229) |
| **Lagomorpha** | *Ochotona princeps* | [ENSOPRG00000014791](http://www.ensembl.org/Ochotona_princeps/geneview?gene=ENSOPRG00000014791) | [ENSOPRG00000006536](http://www.ensembl.org/Ochotona_princeps/geneview?gene=ENSOPRG00000006536) | ENSOPRG00000008167 | - | | [ENSOPRG00000009335](http://www.ensembl.org/Ochotona_princeps/geneview?gene=ENSOPRG00000009335) | | [ENSOPRG00000006712](http://www.ensembl.org/Ochotona_princeps/geneview?gene=ENSOPRG00000006712) | - | - | - | | - | - | [ENSOPRG00000014980](http://www.ensembl.org/Ochotona_princeps/geneview?gene=ENSOPRG00000014980) |
|  | *Oryctolagus cuniculus* | [ENSOCUG00000001555](http://www.ensembl.org/Oryctolagus_cuniculus/geneview?gene=ENSOCUG00000001555) | - | ENSOCUT00000004637 | [ENSOCUG00000017852](http://www.ensembl.org/Oryctolagus_cuniculus/geneview?gene=ENSOCUG00000017852) | | [ENSOCUG00000008833](http://www.ensembl.org/Oryctolagus_cuniculus/geneview?gene=ENSOCUG00000008833) | | - | - | [ENSOCUG00000006758](http://www.ensembl.org/Oryctolagus_cuniculus/geneview?gene=ENSOCUG00000006758) | [ENSOCUG00000000478](http://www.ensembl.org/Oryctolagus_cuniculus/geneview?gene=ENSOCUG00000000478) | | - | - | - |
| **Superorder Afrotheria** | |  |  |  | |  |  | |  |  |  | |  |  |  |  |
| **Hyracoidea** | *Procavia capensis* | [ENSPCAG00000015118](http://www.ensembl.org/Procavia_capensis/geneview?gene=ENSPCAG00000015118) | - | - | [ENSPCAG00000016592](http://www.ensembl.org/Procavia_capensis/geneview?gene=ENSPCAG00000016592) | | .- | | - | - | - | - | | - | [ENSPCAG00000012651](http://www.ensembl.org/Procavia_capensis/geneview?gene=ENSPCAG00000012651) | [ENSPCAG00000000661](http://www.ensembl.org/Procavia_capensis/geneview?gene=ENSPCAG00000000661) |
|  |  |  |  |  |  | |  | |  |  |  |  | |  |  | - |
| **Afrosoricida** | *Echinops telfairi* | ENSETEG00000000360 | [ENSETEG00000004119](http://www.ensembl.org/Echinops_telfairi/geneview?gene=ENSETEG00000004119) | - | -. | | - | | - | - | - | [ENSETEG00000019146](http://www.ensembl.org/Echinops_telfairi/geneview?gene=ENSETEG00000019146) | | - | - | - |
| **Proboscidea** | *Loxodonta africana* | [ENSLAFG00000006295](http://www.ensembl.org/Loxodonta_africana/geneview?gene=ENSLAFG00000006295) | - | ENSLAFG00000022222 | - | | -. | | [ENSLAFG00000015690](http://www.ensembl.org/Loxodonta_africana/geneview?gene=ENSLAFG00000015690) | [ENSLAFG00000026942](http://www.ensembl.org/Loxodonta_africana/geneview?gene=ENSLAFG00000026942) | [ENSLAFG00000009224](http://www.ensembl.org/Loxodonta_africana/geneview?gene=ENSLAFG00000009224) | [ENSLAFG00000032759](http://www.ensembl.org/Loxodonta_africana/geneview?gene=ENSLAFG00000032759) | | - | [ENSLAFG00000022406](http://www.ensembl.org/Loxodonta_africana/geneview?gene=ENSLAFG00000022406) | - |
| **Superorder Xenarthra** | |  |  |  | |  |  | |  |  |  | |  |  |  |  |
| **Pilosa** | *Choloepus hoffmanni* | - | - | - | - | | - | | - | - | [ENSCHOG00000011565](http://www.ensembl.org/Choloepus_hoffmanni/geneview?gene=ENSCHOG00000011565) | - | | - | - | - |
| **Edentata** | *Dasypus novemcinctus* | - | - | - | [ENSDNOG00000025633](http://www.ensembl.org/Dasypus_novemcinctus/geneview?gene=ENSDNOG00000025633) | | - | | - | - | - | - | | - | [ENSDNOG00000012229](http://www.ensembl.org/Dasypus_novemcinctus/geneview?gene=ENSDNOG00000012229) |  |
| **Superorder Marsupialia** | |  |  |  | |  | |  |  |  |  | |  |  |  |  |
| **Didelphimorphia** | *Monodelphis domestica* | - | - | - | [ENSMODG00000003825](http://www.ensembl.org/Monodelphis_domestica/geneview?gene=ENSMODG00000003825) | | - | | - | - | - | [ENSMODG00000009201](http://www.ensembl.org/Monodelphis_domestica/geneview?gene=ENSMODG00000009201) | | - | - |  |
| **Diprotodontia** | *Macropus eugenii* | ENSMEUG00000001831 | [ENSMEUG00000007726](http://www.ensembl.org/Macropus_eugenii/geneview?gene=ENSMEUG00000007726) | ENSMEUG00000004699 | - | | - | | - | - | - | - | | - | - |  |
| **Monotremata** | *Ornithorhynchus anatinus* | - | - | - | - | | - | | - | - | - | ENSOANG00000013737 | | - | - |  |
